# *Neurospora crassa* Light Signal Transduction Is Affected by ROS

**DOI:** 10.1155/2012/791963

**Published:** 2011-10-20

**Authors:** Tatiana A. Belozerskaya, Natalia N. Gessler, Elena P. Isakova, Yulia I. Deryabina

**Affiliations:** A. N. Bach Institute of Biochemistry, Russian Academy of Sciences, 33 Leninsky Prospekt, Moscow 119071, Russia

## Abstract

In the ascomycete fungus *Neurospora crassa* blue-violet light controls the expression of genes responsible for differentiation of reproductive structures, synthesis of secondary metabolites, and the circadian oscillator activity. A major photoreceptor in *Neurospora* cells is WCC, a heterodimeric complex formed by the PAS-domain-containing polypeptides WC-1 and WC-2, the products of genes *white collar-1* and *white collar-2*. The photosignal transduction is started by photochemical activity of an excited FAD molecule noncovalently bound by the LOV domain (a specialized variant of the PAS domain). The presence of zinc fingers (the GATA-recognizing sequences) in both WC-1 and WC-2 proteins suggests that they might function as transcription factors. However, a critical analysis of the phototransduction mechanism considers the existence of residual light responses upon absence of WCC or its homologs in fungi. The data presented point at endogenous ROS generated by a photon stimulus as an alternative input to pass on light signals to downstream targets.

## 1. Introduction

The light perception of fungi is a part of the complex sensory system responding also to changes in the concentrations of nutrient substrates, hormones, temperature shifts, mechanical damage, and so forth, which allows the fungus to adapt its vital functions to environmental changes [1–3]. Fungi use light as a source of information but not as a source of energy.

Light, as all stress agents, increases intracellular concentration of reactive oxygen species (ROS) in fungi [1, 4]. Experimentally detected relationship of developmental processes with the action of factors increasing intracellular ROS concentration indicated that ROS act as signaling molecules regulating physiological responses and developmental processes in fungi [3, 5–7].

Considerable recent attention is focused to molecular mechanisms of ROS signal reception and transduction and modification of gene activity in response to stress factors. 

Absence of biological motility and lack of behavioral responses in fungi led to induction of the synthesis of compounds (especially carotenoids and melanins in the case of light action) that ensure increased resistance to detrimental effects. Another adaptive response is the differentiation of survival structures such as sclerotia and, of course, spores—the copies of genetic material of the organism, well protected from damaging environmental influences.

*Neurospora crassa* has served as a model organism to study light responses in eukaryotic cells for several decades [2, 8–11]. In this organism, various processes of differentiation such as the induction of carotenoid production in mycelia [12], protoperithecial formation [13], phototropism of perithecial beaks [14], perithecial polarity [15, 16], and circadian rhythm [17, 18] are controlled by blue light, which is associated with the generation of ROS [4, 19–21]. Underlying these biological phenomena is the regulation of many *Neurospora* genes by light. Recently, of the 5600 detected genes on a whole genome microarray, approximately 5.6% or 314 responded to a light stimulus by a relatively rapid increase in transcript amount [22].

*Neurospora crassa* uses blue light (350–500 nm) as the primary signal for photoreception. The primary photoreceptor system for blue light in the fungus is the white collar (WCC) complex, a protein complex formed by two proteins WC-1 and WC-2. WC-1 is a protein with a flavin-binding domain and a zinc-finger domain and interacts with WC-2, another zinc-finger domain protein. The WCC complex operates as a photoreceptor and a transcription factor for blue-light responses in *Neurospora*. It represents also a key transcription factor for circadian oscillator [10, 23].

On the other hand it has been shown that manipulation of ROS was a strategy to regulate cell differentiation in *Neurospora crassa* [5, 7, 24, 25]. In order to take a step closer to understanding ROS functions in *Neurospora *differentiation, the present review considers participance of ROS in blue light signal transduction through *N. crassa* WCC complex.

## 2. Light in *Neurospora* Development and Differentiation

After the classic studies performed by Beadle and Tatum in the 1940s, *Neurospora* became a recognized model in genetic and biochemical studies. *Neurospora* is multicellular and produces at least 28 morphologically distinct cell types, many of which are derived from hyphae [26, 27]. The mycelium of *N. crassa* is composed of multinuclear branched hyphae which show apical polar growth. The hyphae are divided into compartments (100–200 *μ*m) by septa, each having a central pore up to 0.5 *μ*m in diameter. The pore is permeable to cytoplasm, nuclei, and mitochondria. The septal pores of *N. crassa* are considered to be functional analogues of gap junctions of animal cells, plasmodesmata of plants, and microplasmodesmata of filamentous cyanobacteria [28]. The diffusional and electric relationships between hyphal cells are local, as it is in other organisms, and involve three or four compartments along the hypha. These relationships appear to be genetically determined and controlled by the gradient of membrane potential between hyphal compartments. They are also controlled by light of the blue-violet spectral area [1, 28].

**Figure 1:**
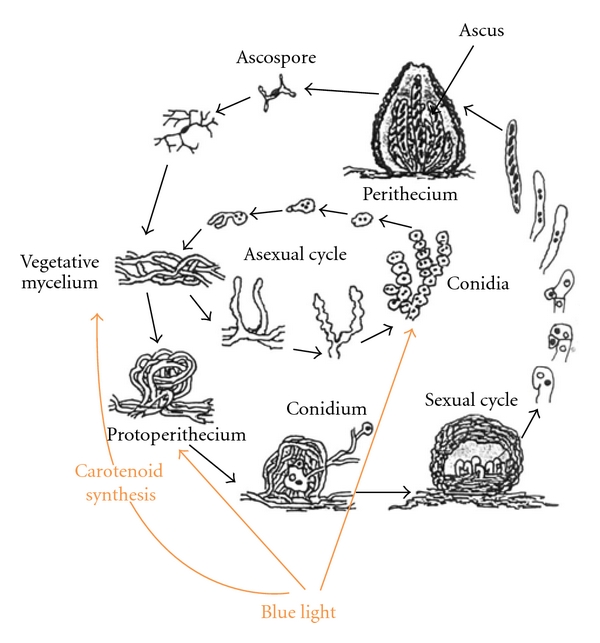
Life cycle of *Neurospora crassa*. Depending on environmental conditions, the vegetative mycelium can undergo the asexual sporulation processes (macroconidiation and microconidiation). It can enter the sexual cycle by forming protoperithecia. Upon fertilization, they initiate development leading to the production of meiotically derived ascospores. Blue light inputs are shown by arrows.

Frequent fusion among hyphal filaments produces a complex hyphal network (the mycelium) [29] and promotes the formation of heterokaryons in which multiple genomes can contribute to the metabolism of a single mycelium. Specialized aerial hyphae are differentiated from vegetative hyphae in response to nutrient deprivation, desiccation, or various stresses, and these form chains of asexual spores (the multinucleate macroconidia) for dispersal [30] (Figure 1). The timing of macroconidiation is controlled by a circadian rhythm, which in turn is modulated by exposure to blue light. Another type of asexual spore, the uninucleate microconidium, is differentiated from microconidiophores or directly from the vegetative hypha [27, 30–32]. Limiting nitrogen induces a type of hyphal aggregation that leads to generation of multicellular female sexual organs (protoperithecia) [32, 33]. Mating is accomplished by chemotropic growth of a specialized female hypha from the protoperithecium toward the male cell (typically a conidium) in a process involving pheromones [34]. Once fertilized, protoperithecia increase in size, darken, and transform into perithecia. The sexual process is followed by a short-term diploid phase. In the perithecia, a fruiting body, black (melanin-containing) ascospores (haploid spores of the sexual cycle) mature for several days after meiosis. Each perithecium comprises 200–400 asci, each containing eight oval mononuclear haploid ascospores. During germination of ascospores, hyphae of vegetative mycelium develop, as in the case of conidia (Figure 1).

The genome of *Neurospora*, comprising 42.9 million bp, has been decoded [35]. The network of the fungus chromosomes includes 527 multigene families containing approximately 10,000 genes. Consistent with the greater biological complexity of filamentous fungi compared to both fission and budding yeast, *Neurospora* possesses nearly twice as many genes as *Schizosaccharomyces pombe* (4,800) and *S. cerevisiae* (6,300). *Neurospora* contains almost as many genes as *Drosophila melanogaster* (14,300), despite the relative developmental complexity of the latter [35]. The *Neurospora* gene complement also displays greater structure complexity than that of the two yeasts.

*Neurospora* can be easily cultured on media of a specific chemical composition. Its development cycle takes one to two weeks. A change of morphologically distinct development phases is easily induced by a change in the composition of the culture medium or other related factors. Quiescent spores germinate to form a haploid vegetative mycelium with hyphae spreading over the substrate at a rate of up to 10 cm/day. Filamentous branching hyphae of the mycelium are approximately 10–20 *μ*m in diameter.

**Figure 2:**
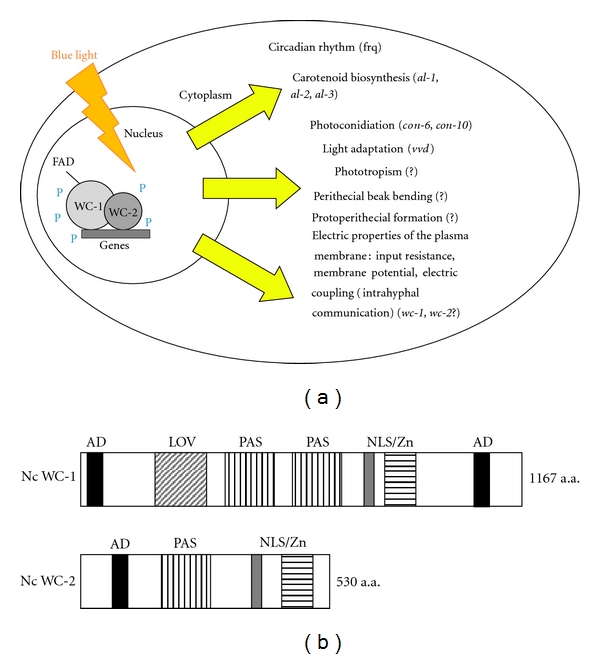
Blue light reception through WCC complex. (a) WCC-mediated gene expression and various light responses in *Neurospora crassa.* (b) Photoreceptor proteins in *Neurospora crassa*. The figure shows two multidomain proteins WC-1 and WC-2 forming photoresponsive WCC complex. WC-1 interacts with WC-2 through PAS (protein-protein interaction) domains. LOV-domain (a specialized variant of the PAS domain) in photoreceptor WC-1 noncovalently binds FAD. The two proteins contain activation domains (ADs), DNA-binding Zn-finger domains, and nuclear localization domains (NLSs).

The effect of light is manifested at different stages of the *Neurospora* life cycle (Figures 1 and 2). Light promotes changes in the electrophysiological parameters of hyphae: the input resistance increases, followed by hyperpolarization of the cytoplasmic membrane [36]. The last phenomenon may be accounted for by regulation of activity of H^+^-ATPase, a plasma membrane proton pump [37].

Changes in these parameters are transient, and their values subsequently return to the initial level. Illumination also affects the intercellular communication mechanism (electric-bond coefficient) or, in other words, the rate of diffusion of ions between interseptal hyphal areas. It can be assumed that light-dependent changes in electrophysiological parameters are part of the energy cooperation system in interseptal hyphal areas, which allows the fungus to more effectively supply energy in the form of membrane potential for membrane transport in the apical compartment of growing hyphae [28]. The photoreceptor mutant *white collar *1 (*wc-1*) has a lower constitutive membrane potential, disrupted intrahyphal communication mechanisms, and it lost all the blue-light induced electrical reactions: a transitive increase of input resistance and membrane potential [28]. Thus changes in the electrical properties of the *N. crassa* plasma membranes upon the light action appear to be controlled via WCC complex (Figure 2).

**Figure 3:**
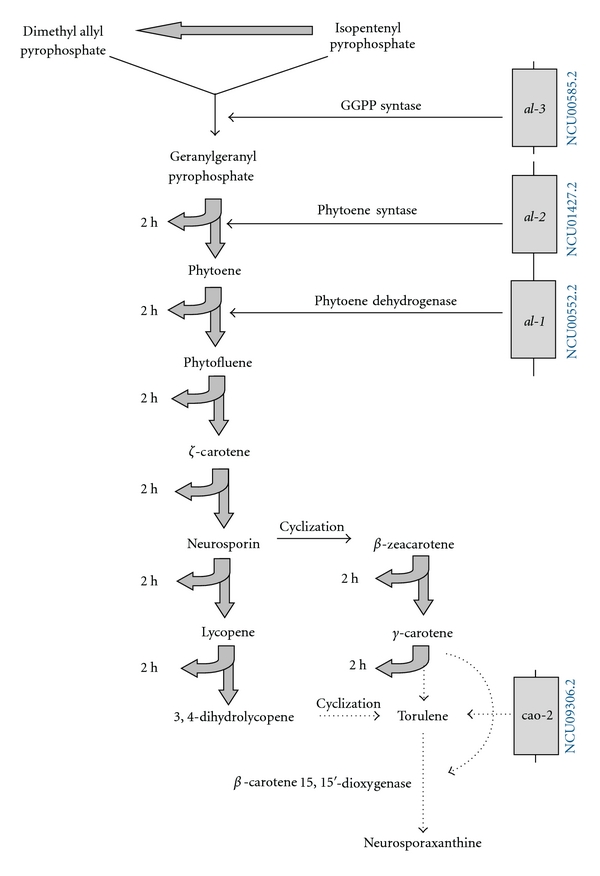
The enzyme pathway of carotenoid biosynthesis in *Neurospora crassa*. NCU numbers of light-regulated genes are shown. The figure is modified from Uspekhi Biologicheskoi Khimii [1].

Light induces the expression of genes *albino* (*al-*1*, al-*2, and *al-*3) involved in carotenogenesis in hyphae and, as a result, the accumulation of neurosporaxanthine and other pigments imparting orange color to the mycelium (Figure 3) [38, 39]. Carotenogenesis in conidia, in contrast to mycelium, has a constitutive nature. The synthesis of carotenoids—the quenchers of oxygen-excited states and the inhibitors of free radical processes—is regarded as a means of cell defense against light-induced damage.

Two light-regulated phenomena, the electrogenic transport function of membrane and accumulation of carotenoids in the cell, are apparently physiologically related. In the *nap *mutant, damage of the proton pump which consumes as much as 50% of intracellular ATP caused an increase in the content of ATP and utilization of its energy in other metabolic processes (including the synthesis of precursors of carotenoids); as a result, the synthesis of pigments increased [40].

Light also affects some mycelial enzymes. For example, illumination increases the degree of phosphorylation of nucleoside diphosphate kinase [16], activates cAMP phosphodiesterase [41], and changes the activity of molecular forms of NAD^+^-kinase [42]. In addition, light changes the inactive (reduced) form of nitrate reductase into the active (oxidized) state [43]. Photoreactivation with near ultraviolet light (UV-A) of DNA molecules damaged by more shortwave radiation occupies a special place. This phenomenon is based on DNA photolyase-catalyzed cleavage of C–C bonds between neighboring pyrimidine bases [44].

As mentioned above, differentiation of reproductive structures is controlled by a complex of external signals whose effect is regulated by the cell, with light playing a key role in this mechanism [45]. Exhaustion of a nutritive substrate is a necessary condition of differentiation. Some effects (e.g., carbon starvation or mycelium drying) promote rapid conidiation, with light additionally stimulating this process [45]. Nitrogen starvation induces the formation of protoperithecia and simultaneous (yet less active than in carbon starvation) conidiation. Unlike carbon starvation, in nitrogen starvation light inhibits conidiation and simultaneously stimulates the formation of protoperithecia [46]. In other words, under these conditions light determines the selection of either the sexual or asexual development pathway (Figure 1). Perithecia occurring during the sexual cycle are also sensitive to light, which induces their polarity (i.e., formation of a so-called beak at one end of the perithecium, which, in turns, exhibits positive phototropism) [14] (Figure 2).

Light also affects the circadian rhythm endogenous sensor function. Conidia are formed with a certain periodicity on the mycelium that spreads over the substrate surface, which leads to the occurrence of spatially separated sporulation zones. Pulse illumination changes rhythm parameters, and constant illumination suppresses manifestations of rhythmicity [11].

## 3. Light Effects Are Accompanied by Formation of ROS

All of the environmental stresses triggering *N. crassa* differentiation are apparently sources of ROS [24, 25, 47]. Among these factors are ionizing radiation (alpha, beta, gamma, and X-ray beams), UV radiation (far 200–290 nm, medium 290–320 nm, and near 320–420 nm), and visible light. ROS appear to mediate blue light effects in cells, but the sources of ROS and their respective roles in the cellular response to blue light are not completely understood. Direct evidence of ROS formation under light on various objects is given hereinafter.

High-fluence blue light can induce H_2_O_2_ generation at both the plasma membrane and the chloroplast of *Arabidopsis. *The high-fluence blue light-induced H_2_O_2_ generation can be abolished by the administration of the H_2_O_2_-specific scavenger catalase and other antioxidants or by the addition of diphenyleneiodonium, which is an NADPH oxidase inhibitor, and the blocker of electron transport chain dichlorophenyl dimethylurea [48]. The generation of O_2_^−∙^—(by the coleoptile tip of Sorghum bicolor and wheat (*Triticum vulgare*) was augmented upon illumination with blue light. Various thiol blockers caused powerful inhibition of blue light induced O_2_^−∙^ generation [49]. Blue light increased intracellular ROS equally in both normal human epidermal keratinocytes and oral squamous cell carcinoma. Blue light-generated ROS suppress cellular mitochondrial activity. However, the identity of blue light targets that mediate these changes remains unclear [21]. In addition, it was found that acute exposure of keratinocytes to both UVA and UVB results in activation of NOX and generation of ROS [50–52]. These studies suggest that a rapid activation of NOX by UV irradiation in these cells may have a distinct physiological importance. How irradiation activates NOX is not totally understood [47]. Thus UV-blue light effects on various organisms provide ROS formation inside the cells.

## 4. Intracellular Sources of ROS in Fungi

ROS are formed in fungi in the course of metabolic activity. The involvement of oxygen in metabolic processes in fungi is coupled to its activation and formation of number of highly reactive compounds such as (O_2_^−∙^), hydrogen peroxide (H_2_O_2_), and OH^∙^. In addition to the respiratory chain, as an intermediate product ROS are generated in reactions with involvement of xanthine oxidase, microsomal monooxygenases, lipoxygenase, and, as a result, of autooxidation of thiols, flavins, quinones, and catecholamines, as well as the reduction of xenobiotics [53].

Certain intracellular enzymes producing ROS cannot be ruled out. These include, first of all, NADPH-oxidases (NOX), specifically producing ROS and playing a significant role in growth and differentiation of *Neurospora crassa* [54–56]. It is known that specific enzymes, such as NOX, produce ROS to regulate different cellular functions, including growth, cell differentiation, development, and redox-dependent signaling [47, 54–56]. The fact that NOX regulate developmental processes in different microbial eukaryotes suggests that ROS regulate cell differentiation, and that this is a ROS ancestral role conserved throughout the eukaryotes [47]. Enzymes belonging to the NOX family produce O_2_^−∙^ in a regulated manner. It has been shown in *N. crassa* that NOX-1 and NOX-2 are both involved in different aspects of growth and development; a single regulatory subunit, NOR-1, an ortholog of the mammalian NOX-2 regulatory subunit gp67 (phox), is regarded for the function of both NOX. *N. crassa* NOX-1 elimination results in complete female sterility, decreased asexual development, and reduction of hyphal growth. The lack of NOX-2 did not affect any of these processes but led instead to the production of sexual spores that failed to germinate, even in the presence of exogenous oxidants. These results indicate a link between NOX-generated ROS and the regulation of growth [55].

It was revealed that NO^*∙*^ synthase participated in asexual spore development of *N. crassa* and in differentiation of other fungi [57]. Glyoxal oxidase appeared to be involved in differentiation of phytopathogenic fungi [58, 59].

## 5. ROS in *Neurospora* Development and Differentiation

### 5.1. Changes in ROS Concentration and Differentiation in Fungi

High reactivity of ROS is responsible for oxidation of proteins, lipids, and nucleic acids. Consequently, systems defending against ROS by repair or resynthesis of damaged molecules are present in the cell. Nevertheless, impairment of intracellular redox status, as a result of an increase in generation of oxygen radicals exceeding the cellular capacity to neutralize them, can generate a hyperoxidation state (oxidative stress). As distinct from growth and differentiation state, oxidative stress is an unstable one, and elimination or partial inhibition of intracellular antioxidant systems may cause cell death [5, 7]. Intracellular ROS increase is accompanied by the cessation of growth, and it provokes morphological changes leading to cell adaptation to changes in life conditions as well as the decrease in intracellular oxidants. Numerous experimental data support the relationship of differentiation triggering processes with an increase in intracellular ROS [5, 60–63]. Just so, in the myxomycete *D. discoideum* an increase in intracellular O_2_^−●^ as well as extracellular one provoked aggregation of myxamoebae and subsequent differentiation, and the aggregation process was prevented by O_2_^−●^ scavengers together with an increase in expression of genes controlling antioxidant defense systems (ADS) [62].

H_2_O_2_ is considered as one of the most important metabolites in all respiring cells. H_2_O_2_ provoked global changes of gene transcription, including the ADS genes, in *A. nidulans* [60], as well as sclerotial differentiation in *Sclerotium rolfsii* [63], increased expression of genes of carotenogenesis in *N. crassa* [64], and promoted transition to filamentous growth in *U. maydis* and development of its pathogenicity [59]. It is known that sclerotial differentiation in *S. rolfsii* is coupled to H_2_O_2_ generation inside the cell. Its concentration increased under the action of light and iron ions [63].

OH^●^ formed on the interaction of transition metals with H_2_O_2_ was inhibited by such scavengers as dimethylsulfoxide, phenylthiourea, p-nitrosodimethylaniline, ethanol, and benzoate, which suppress sclerotial differentiation in *S. rolfsii* [65]. Sclerotial differentiation was similarly inhibited by antioxidants (ascorbic acid, *β* carotene) [66, 67]. It was shown that O_2_^−●^ increased cleistothecium differentiation in *A. nidulans* [68], while NO^●^ promoted fruit body development in *F. velutipes* [69].

At the onset of different stages of *N. crassa* macroconidia differentiation (aggregation of hyphae, aerial hyphae formation, differentiation of macroconidium), a spontaneous, low-level chemiluminescence was detected enhanced by lucigenin and/or luminol, indicative of an increase in level of intracellular oxygen radicals. Antioxidants abolished chemiluminescence and stopped differentiation, which supports the formation of ROS ahead of every stage of fungal development [70]. Thus ROS formation is essential for differentiation of *N. crassa* as well as development of other fungi.

### 5.2. Changes in Fungal Cell Metabolism under ROS Action

An increase of oxidant level inside the cell inevitably causes the oxidation of organic molecules. It has been shown that differentiation of sclerotia on the mycelium of *S. rolfsii* was accompanied by lipid peroxidation [70]. Light and Fe^2+^ enhanced lipid peroxidation as well as the intensity of sclerotium formation [63], and lipid peroxides and aldehyde degradation products inhibited many proteins, affected cell differentiation and proliferation, and might promote apoptosis [71].

Oxidation of sulfhydryl groups in proteins upon ROS action promotes a change in activity of some enzymes. As an example, decrease in glycolytic enzymes and decline of protein synthesis enzymes have been observed, coupled to cessation of growth [72, 73].

Oxidative stress was accompanied by cessation of growth and severe metabolic changes directed towards decrease in primary metabolites (acetate, glucose) and synthesis of compounds participating in cell protection, for example, carotenoids, melanins, proline, and polyols [3]. Trehalose is of fundamental importance in defending yeast cells in oxidative stress [74]. At the start of separate steps of macroconidium differentiation in *N. crassa*, mass protein oxidation and their subsequent degradation [75], release of iron ions upon oxidation of [Fe-S] clusters of enzymes, oxidation of intracellular NADP and NADPH, glutathione oxidation, glutathione disulfide excretion to the extracellular medium [76], synthesis of antioxidant enzymes [7, 77], and ROS-dependent chemiluminescence [24] were the experimental evidence of hyperoxidant state. An increase in protein carbonylation by ROS has been observed in different species of mycelial fungi:* Mucor racemosus*, *Humicola lutea*, *F. oxisporum*, *A. solani*, *Cladosporium elatum*,* Penicillium chrysogenum*, *P. brevicompactum*, *P. claviforme*, *P. roquefortii, A*. *niger, A. argilacceum, A. oryzae, *and* N. crassa* [78].

A comparative study of the changes in the components of the ADS, the activity of superoxide dismutase (SOD) and catalase and the level of extractable SH-groups, during the growth of wild-type and *N. crassa* mutants (*white collar-*1 and *white colar-*2) showed that oxidative stress developing during spore germination and upon the transition to a stationary growth phase was accompanied by an increase in the level of extractable SH-groups and SOD activity in all the strains, whereas the total catalase activity decreased during growth. However, in contrast to the wild-type strain, the activity of the catalase in the mutant strains *wc-*1 and *wc-*2 slightly increased upon the transition to the stationary phase. In the *wc-*2 mutant, SOD activity and the level of extractable SH-groups in the exponential growth phase were always lower than those in the wild-type and *wc-*1 strains [79]. As in previous works [5, 7, 75, 76], our data pointed to formation of ROS upon transition to interchangeable phases of development. Moreover, the data revealed that mechanisms of inactivation of increased intracellular ROS, developing during spore germination and entry into the stationary growth phase, distinguished *wc-*1 and *wc-*2 mutants from the wild strain [79]. These data prompted us to pay a closer attention to mechanisms of blue light signal transduction through WCC and to the role of ROS in this process.

## 6. Photoreceptor Complex, Other Photoreceptors, and Other Signal Transduction Pathways

### 6.1. Photoreceptor Complex WCC

The main blue-light responses in *Neurospora* include induction of sporulation and sexual development, induction of carotenoid synthesis in mycelium, and the regulation of circadian clock. All of the mentioned processes require the products of *white collar* 1 (*wc-*1) and *white collar* 2 (*wc-*2) genes-GATA zink finger family members [80]. WC-1 is the product of the *wc-*1 gene, a protein with a Zn-finger, two PAS domains involved in protein-protein interactions, a putative transcriptional activation domain, a nuclear localization signal, and a chromophore-binding domain [81] (Figure 2). The chromophore binding domain binds the flavin chromophore FAD allowing WC-1 to act as a photoreceptor [82, 83]. The WC-1 flavin-binding domain (LOV-light, oxygen, voltage) has been described in other photoreceptor proteins, most notably in plant phototropins [84]. The primary photochemical event in phototropins is the formation of a flavin-cysteinyl adduct at a cysteine of the LOV domain [85], suggesting that WC-1 activation occurs through the formation of a light-dependent flavin-cysteinyl adduct [86].

WC-2 is the product of the *wc-*2 gene, a protein with a zink finger, a single PAS domain, a putative transcriptional activation domain, and a nuclear localization signal [87] (Figure 2). WC-1 and WC-2 interact through the PAS domains to form a WCC complex [88–93]. In the WCC complex, WC-1 is the limiting factor while WC-2 is in excess [89, 93]. WCC binds the promoter of light inducible genes [81, 82, 94–96]. Light causes a decrease in the mobility of the WCC complex bound to the promoter, suggesting a light-dependent aggregation of WCC complexes [82, 96].

The WCC proteins are present in the dark [92, 93, 97] and are preferentially located in the nucleus although WC-2 is also observed in the cytoplasm and is more abundant than WC-1 [92, 95, 97, 98]. Nuclear localization of either WC-1 or WC-2 is not affected by light and is not altered by mutations in *wc-*2 or *wc-*1, respectively, indicating that nuclear localization does not require a complete WCC complex [97].

Microarray analysis showed that the expression of 314 genes responded to the light stimulus by increasing transcript levels [22]. Most of the identified genes (92%) were either early (45%), with peak expression between 15 and 45 minutes, or late (55%), with the induced expression peaking between 45 and 90 minutes after lights on [1, 99]. Genes related to the synthesis of photoprotective pigments (7.1%), vitamins, cofactors, and prosthetic groups (4.7%), secondary metabolism (4.7%), DNA processing (6.3%), cellular signaling (5.5%), and environmental sensing and response (1.6%) were found enriched in the early light response. In contrast, genes involved in carbohydrate metabolism (20%), oxidation of fatty acids (1.9%), and oxygen detoxification reaction (2.5%) were found enriched in the late light response. Within the early group were several transcription factors most of which show mutant phenotypes during development. Transcription factor SUB1 is required for efficient transduction of light signals to the most of late light response genes [22, 99].

Gene photoactivation is transient. After further light exposure, WC-1 is phosphorylated [93, 96, 97, 100] leading to exclusion of the WCC complex from the promoter and the end of gene transcription.

The protein VIVID, an additional *N. crassa* photoreceptor, is a flavoprotein and serves as a fungal blue light photoreceptor for photoadaptation [22]. VIVID (VVD) is a small protein (186 amino acids) with a single LOV domain functioning downstream of the WCC to regulate negatively the light responses initiated by the WCC [22, 86, 101–104]. The induced VVD protein accumulates in the nucleus and physically interacts with WCC to regulate photoadaptation by repressing WCC activity in constant light. The kinetics of photoadaptation is predominantly regulated by the amount of VVD protein in the system [105].

The excluded WCC complex is dephosphorylated and partially degraded, probably through an interaction with the protein kinase C (PKC). Since protein phosphatase 2A participates in the dephosphorylation and activation of the WCC complex in vivo [95], it is possible that this enzyme is also involved in the dephosphorylation of the WCC complex after light exposure. After a certain period in the dark the WCC complex, probably with the addition of newly synthesized WC-1 and WC-2, is ready for gene photoactivation again.

The amount of WC-1 and the kinetics of the light-dependent phosphorylation is altered by the presence of a mutant form of WC-2 suggesting that WC-2 is necessary to sustain the transiency and magnitude of WC-1 phosphorylation [93, 97, 106].

In *Neurospora*, the photoreceptor complex WCC serves as an exogenous regulator of the circadian clock which is an important recipient of light information (Figure 2). The circadian clock controls the program of *Neurospora* development [107, 108]. When cultured in the dark in the absence of external signals, the fungus periodically (with an approximately 21.5-hour period in the case of wild-type cells) switches from mycelial growth to conidiation. VVD has been shown to take part in regulating various circadian clock properties, most likely through its effects on the WCC, including gating of light input to the clock [101], maintenance of the clock during the light phase [102, 103], and temperature compensation of the circadian phase [104].

Thus, the first fungal photosensor identified was White collar-1 (WC-1) of *N. crassa* [108], and this system has been extensively studied with emphasis on the circadian clock of this fungus and how it is regulated by the WC-1 and WC-2 proteins, the clock protein FRQ and interacting factors [109, 110]. More recently, WC-1 homologues have been identified in basidiomycetes [111, 112] and zygomycetes [113, 114] as well as other ascomycetes [115, 116]. This information extends the function of WC-1 homologues in photosensing across the fungal kingdom.

### 6.2. Other Photoreceptors

The *Neurospora *genome contains genes for additional photoreceptors, including a cryptochrome gene (*cry*), an opsin gene (*nop* 1), and two phytochrome genes (*phy* 1 and *phy *2), but their function in *Neurospora* photobiology remains mostly unknown [26, 35].

It has been shown recently that the activity of the WCC is negatively regulated by the photoreceptors CRY-1, NOP-1, and PHY-2, presumably through the light-dependent activation of a putative repressor of the WCC. It is possible that each photoreceptor may activate an independent repressor of the WCC [117].

The regulation by secondary photoreceptors of the WCC may modify the activity of some genes, as it was observed for *con-*6, *al-*1, and *vvd.* It has been suggested that a light-dependent repressor of the WCC may be a general feature of light reception in *N. crassa* [117].

A major regulator of conidiation in *Aspergillus nidulans* is the product of the gene *veA*. VeA is preferentially located in the nucleus in cells grown in the dark, which is consistent with the role of VeA as a repressor of light-dependent processes [118], and the VeA protein interacts in a complex with other regulatory proteins for the regulation by light of development and secondary metabolism [119]. The *Neurospora* ortolog *ve-1* encodes a protein Ve-1. A pronounced reduction in light-dependent carotenoid accumulation (threefold) was observed in the *ve-*1 strain suggesting that the putative regulatory Ve-1 protein is required for full photocarotenogenesis in *Neurospora* [117]. 

### 6.3. The Complexity of *Neurospora* Light Sensing Cascade

It cannot be ruled out that there are possibly multiple intertwined pathways in the mechanism of photosignal transduction. The analysis of promoters of approximately 20 light-inducible genes did not reveal any common cis-acting elements (i.e., DNA sequences that are recognized by light-dependent transcription factors). The situation is additionally complicated by the fact that there are light-dependent genes whose expression is not mediated by the functional proteins WC-1 and WC-2 [87, 120].

An additional complicating factor may be chemical modification (enzymatic methylation) of these sequences. Although the genome of *Neurospora *is methylated fairly weakly, it is known that the level of methylation may undergo changes in the course of ontogeny [121]. It should be noted that methylation plays an important role in ontogenetic photoregulation. There are grounds to believe that the WCC complex is involved in the regulation of DNA methylation, the level of which determines the light-dependent selection of either sexual or asexual development by the fungus. It was shown that 5-azacitidine, an inhibitor of DNA methylation, suppressed photoinduced formation of sexual structures (protoperithecia) and simultaneously abolished the inhibitory effect of light on conidiogenesis [46, 122].

It should be noted that illumination induced a rapid and transient (30–600 s) decrease in the cAMP content in *Neurospora* mycelium [123, 124] as a result of increase in the activity of cAMP phosphodiesterase [41]. The treatment with 3-isobutyl-1-methylxanthine, an inhibitor of phosphodiesterase, as well as addition of exogenous cAMP, inhibited the cell response to illumination (expressed as the synthesis of carotenoids), whereas a decrease in the cAMP level, observed in some mutants, was accompanied by induction of carotenogenesis in the dark. Exogenous cAMP completely inhibited photoinduction of expression of the genes *al-*1*, al-*2, *bli-*3*, bli-*4*, ccg-*2*, con-*8, and *con-*10. It can be assumed that the effect of cAMP may be implemented at the transcriptional level via the cyclic AMP response element (CRE) in the promoters of photoinducible genes [125]. In addition, it cannot be excluded that photoregulation influences the change in phosphorylation of WC proteins that is catalyzed by cAMP-dependent protein kinase. The involvement of cAMP pathway in blue light signal transduction, possibly with the involvement of RAS protein, was mentioned in several investigations on *N. crassa* [22, 126, 127] and *Trichoderma atroviride* [128]. Possibility of ROS participation in blue-light signal transduction makes the regulatory networks of the *Neurospora* light-sensing cascade far more complicated.

## 7. ROS Affected WCC Signal Transduction

According to previous studies with various fungi an assumption was produced that intracellular redox state and light-induced carotenogenesis were related processes [129, 130].

Sure enough, some experimental data consider participance of ROS in blue light signal transduction through WCC complex in *N. crassa*. It was shown that illumination of *Neurospora *mycelium under O_2_-enriched air increased transcript level of *al-*1 encoding phytoene dehydrogenase. It also highly enhanced carotenoid production in the mycelium [131]. These results suggest that increased ROS, under oxygen enriched air, could increase light-induced carotenoid production and might act as a controlling factor in the WCC-signaling cascade, because the light-induced expression of *al-*1 mRNA depends on WCC complex function [64]. This assumption was supported by the fact that *sod-*1 mutant, with a defective Cu, Zn-SOD showed accelerated light-dependent induction of carotenoid biosynthesis in the mycelium compared to the wild type [131]. In *N. crassa*, catalase-3-deficient mutants showed increased carotenoid production in colonies under illumination [25]. It was found that menadione treatment of *Neurospora wc* mutants restored circadian conidiation in *N. crassa* [127].

Thus intracellular ROS apparently enhance several light-induced responses in *N. crassa*. They increase the blue-light action but apparently do not trigger WCC-induced blue-light responses.

It is well known at present that increase in intracellular ROS is accompanied by activation of intracellular ADS specific to developing ROS [132]. Antioxidant enzymes (SOD and catalase) in wild type and *wc-*1 and *wc-*2 mutants of *N. crassa* responded differently to various stress factors (oxygen, light, temperature increase) which rise intracellular ROS in cells. Menadione treatment provided SOD increase in the wild type. The enzyme activity did not change in WCC-mutants (*wc-*1 and *wc-*2) [133]. Protein carbonyls (oxidative stress marker) showed a double increase in the wild type (but not in the mutants) as a result of menadione treatment [133]. No increase in SOD activity as well as no rise in protein carbonyls was revealed in WCC mutants thus demonstrating high resistance of the mutant strains to menadione.

It should be noted that a high increase in catalase activity was found only in WCC mutants (*wc-*1 and *wc-*2) upon action of stress agents increasing intracellular ROS [134]. Increase in catalase activity in the mutants upon H_2_O_2_ treatment points to H_2_O_2_ signal transduction independent of WCC. Thus WCC apparently participates in environment signal transduction forming intracellular O_2_^−●^ in the wild type. Lack of SOD activity increase upon stress agents, including light, and high resistance of WCC mutants to menadione, points apparently to some other systems preventing intracellular O_2_^−●^ formation in *wc-*1 and *wc-*2 [134].

As it has been mentioned before, each morphogenetic step of *N. crassa *conidiation was preceded by NAD(P)(H)/NAD(P) and GSH/GSSG redox imbalance [76]. Generation of singlet oxygen was observed during germination of *N. crassa* conidia [135]. Exposure of fungal cells to oxidative stress results in the modulation of various signaling pathways. Oxidation and reduction of protein thiols are thought to be the major mechanisms of ROS integration into cellular signaling pathways. It has been shown that incubation in air (increased intracellular ROS) provided a significant protein disulfide increase only in the *N. crassa *wild type mycelium particularly under light treatment. A decrease in the formation of disulfide bonds in the proteins of *wc-*1 and *wc-*2 mutants (as compared with the wild type strain) was recorded [134]. It can be assumed that at least one ROS signal transduction pathway may be controlled by the WCC.

The main intracellular source of ROS is the mitochondria respiratory chain. Comparative analysis of respiratory activity in the *N. crassa* wild type and its photorecepror complex mutants (*wc-*1 and *wc-*2) revealed high cyanide-resistant respiration in the mutant strains under glucose oxidation pointing to the increased activity of alternative oxidase in the mutant strains. This fact was confirmed by inhibitory analysis [136]. Transfer of electrons through alternative oxidase is not coupled with ATP synthesis. Alternative oxidase prevents autooxidation of electron carriers under ROS increase [137]. It can be assumed that antioxidant defence in WCC mutants is performed using catalase and alternative oxidase.

The data presented show that signal transduction via WCC complex enhances oxidative stress in *Neurospora* cells. In the WCC mutants—*wc-*1 and *wc-*2—no experimental evidence of oxidative stress was revealed. On the other hand, alternative signal transduction pathways apparently functioned. The fact is confirmed by increase in catalase and alternative oxidase levels in *wc-*1 and *wc-*2 mutants. It can be assumed that accomplishment of blue-light responses through WCC-complex in *Neurospora* cells is coupled to oxidative stress.

## 8. Conclusion

Light signaling pathways and circadian clock have profound effects on behavior in most organisms. *N. crassa* is eukaryotic model for light responses and circadian clock. Sequence and functional orthologs of WC-1 and WC-2 and most of the other light signaling components are widespread among the fungal kingdom. Recent studies have demonstrated that WC-1- and WC-2-like molecules in various fungal species play an essential role in mediating light signals from the Ascomycota, Basidiomycota, and Zygomycota phyla [8–10, 138, 139].

Successful work on the WCC in *Neurospora* has led to fundamental breakthroughs in understanding photobiology in other fungi. While many of the downstream genes regulated by the WCC are not well studied or are uncharacterized, most of them have homologs in plants and mammals. Unfortunately even after extensive research, little is known about mechanisms that directly link photoreceptor activation to signaling pathways eliciting light responses. Proteomic analysis across human, yeast, and bacterium has raised that the cellular stress response can be characterized by the induction of a limited number (300) of highly conserved proteins [140]. It is noteworthy that among the 44 proteins with known functions, 40% of them are related to regulation of the intracellular redox status. It is noticed that increased reactive oxygen species (ROS) generation seems to be a common response in fungal organisms exposed to stresses; thus, redox regulation in fungal cells may represent a second messenger system that is upstream of the fungal stress signaling network. It cannot be excluded that reactive oxygen species generated by a photon stimulus might provide a transcriptional response through redox signaling pathways or serve as an input pass on some extra signal transduction systems to downstream targets correcting for the complexity of *Neurospora* light sensing cascade.
